# A Cross-Sectional Audit of Nutrition and Health Claims on Dairy Yoghurts in Supermarkets of the Illawarra Region of New South Wales, Australia

**DOI:** 10.3390/nu13061835

**Published:** 2021-05-27

**Authors:** Sam-Reith S. Wadhwa, Anne T. McMahon, Elizabeth P. Neale

**Affiliations:** 1 School of Medicine, University of Wollongong, Wollongong, NSW 2522, Australia; srsw710@uowmail.edu.au (S.-R.S.W.); amcmahon@uow.edu.au (A.T.M.); 2 Illawarra Health and Medical Research Institute, University of Wollongong, Wollongong, NSW 2522, Australia

**Keywords:** food standards code, dairy yoghurts, nutrition claims, health claims, claim compliance, nutrition profiling, health star rating

## Abstract

Health and nutrition claims are used by consumers to guide purchasing decisions. In consequence, monitoring and evaluation of such claims to ensure they are accurate and transparent is required. The aim of this study was to investigate the use of nutrition and health claims on dairy-yoghurt products within select Australian supermarkets and assess their compliance with the revised Food Standards Code (FSC). Nutrition, health, and related claims on yoghurt products were assessed in a cross-sectional audit of five supermarkets in the Illawarra region of New South Wales. Claim prevalence, type, and compliance were assessed and products were compared against current rating measures. A total of *n* = 340 dairy yoghurt products were identified. Most products (97.9%) carried at least one nutrition and/or health claim, with nutrition-content claims (93.9%) the most prevalent. Most products (*n* = 277) met the nutrient profiling scoring criterion; while 87.9% of products did not carry the health star rating. Almost all claims surveyed (97.4%) were compliant with the FSC. Health and nutrition claims are highly prevalent across yoghurt categories, with the majority of these compliant with regulations. The ambiguity surrounding the wording and context of claims challenges researchers to investigate consumers’ interpretations of health messaging within the food environment.

## 1. Introduction

Health and nutrition claims are a key component of the food environment, utilised by food companies and manufacturers as a way of informing consumers and influencing purchasing behaviour [1,2]. These claims have been shown to have varying influences on a consumer’s ability to navigate the food environment and make informed health choices [3,4,5,6]. As a major purchasing point for food products, supermarkets are a direct channel between consumers and their respective food environments [7,8,9], reinforcing the need for accurate and coherent health information within this space. 

In Australia and New Zealand, health, nutrition, and related claims are regulated by the Food Standards Code (FSC) [10], specifically Standard 1.2.7, which outlines the types of claims that can be made and the conditions under which they can be made. Under Standard 1.2.7, three types of claims can be made: content claims, general-level health claims, and high-level health claims [10]. Furthermore, in order to make either general or high-level health claims, products must meet the nutrient profiling scoring criterion (NPSC) [11], which categorises foods according to their nutritional composition. Since the revision of the code in 2013 and its subsequent enforcement in 2016, investigation into compliance of food packaging claims in Australia has been limited [12]. While the code has enhanced the ability of manufacturers to promote ingredients and products such as fruit and vegetables [13], investigations into prominent supermarket items such as breakfast cereals [14], muesli bars [15], and ultra-processed foods [16] have found varying proportions of claims not compliant with the FSC. Such noncompliant claims can not only lead to legislative and production implications for food manufacturers, but also potentially mislead consumers’ food practices and behaviours [6,7]. Given the differing levels of compliance observed, it is important that a range of food categories are investigated in order to quantify the use of nutrition and health claims across the food supply. 

Yoghurt is a core dairy food and source of calcium contributing to bone and muscle health, as well as blood and hormonal function [17,18,19,20,21]. Despite these positive health implications, and correlations between health and nutrition claims on yoghurt packaging and heightened consumer desirability and purchasing behaviour [1], research into the quantity and compliance of health, nutrition, and related claims on yoghurts in Australia is limited, pre-dating the changes to the FSC [22,23]. This presents the potential for misinformation within the food environment, resulting in likely misguided consumer choices as well as diminishing the capacity of health professionals, such as dietitians, to decipher and convey product information effectively [24,25,26]. An updated investigation of nutrition and health claims made on yoghurt products and their compliance with regulations is therefore needed. 

The aim of this study was to investigate the use of nutrition, health, and related claims on yoghurt products in Australian supermarkets, and assess the compliance of these products with the revised FSC.

## 2. Materials and Methods 

This study involved a cross-sectional audit of nutrition and health claims made on yoghurt products available in supermarkets in the Illawarra region of New South Wales, Australia.

### 2.1. Selection of Supermarkets

Supermarkets were selected based on market share, socioeconomic status, and previous Illawarra supermarket audits [14,27]. The 2018 Supermarket and Fresh Food Currency Report by Roy Morgan indicated that the Woolworths Group (34.0%), Coles Group (27.6%), and Aldi (11.4%) were the top three Australian supermarket chains in terms of market share [28]. As Woolworths currently holds the greatest share [28], three stores were selected (Unanderra, Bulli, and Shellharbour) for the audit. The location of stores was selected based on varying levels of socioeconomic status [29] and previous audits [14,27] in order to obtain equitable [30] and detailed data relating to yoghurt products in the region. Coles Wollongong and Aldi Wollongong were also selected for analysis due to their significant market share [28] and varied product selection [31,32].

### 2.2. Pilot Study

Prior to full data collection, a pilot study was conducted across two days in January 2020 to assess different data collection methods, and the approximate quantity of yoghurt products available in selected supermarkets. A list of yoghurt products was developed prior to the investigation using Coles’ [33] and Woolworths’ [34] online stores. The keywords “yoghurt” and “fermented dairy products” were searched in each of the online websites in order to develop a comprehensive product checklist prior to pilot data collection. In cases where duplicate items were found, only the Woolworths item was included, as it was the first store piloted. Additions were made to the checklist if products not included on the website were found during a physical review of the supermarket. As Aldi did not have an extensive online platform [31], it was excluded from the pilot study. 

Coles Wollongong and Woolworths Unanderra were assessed during the pilot study, with approximately 310 different yoghurt products found between the stores. Due to Aldi’s narrow product range and lack of preference for home-brand products [35] it was estimated that the chain had approximately 50 different yoghurt varieties in addition to those found in Coles and Woolworths. Items found during the pilot study included: yoghurt tubs and pods, yoghurt pouches, and dairy-free yoghurt varieties. From the investigation and assumptions, it was estimated that 330–380 different yoghurt products would be available for analysis. 

### 2.3. Data Collecion 

Following the pilot study, data collection for the supermarket audit took place over five days in February 2020. Letters were sent to each store manager prior to data collection informing them of the process to be undertaken within the store and the scope of the study. Permission was also sought on the day of data collection from the store manager and was granted. 

For the purpose of the audit, yoghurt products were classified into 11 categories in accordance with the AUSNUT 2011–13 food and dietary supplement classification system for yoghurts [36], with an additional two categories (“children’s yoghurt” and “yoghurt pouches”) included after consultation with the research team and pilot findings (Table 1). Products that did not appear within the AUSNUT Classification System’s yoghurt categories, such as frozen yoghurts and infant yoghurt/custards (defined as those aimed at children aged less than one year of age [37]) were excluded from analysis. In addition, as the focus of this research was on dairy yoghurt (as defined by AUSNUT 2011-13); nondairy-based yoghurt products such as coconut yoghurt were not included in this project. This aligns with the categorisation of these products as “milk substitutes” according to the AUSNUT classification system, and as a separate food group to yoghurt in the FSC [10]. Furthermore, items not available in analysed supermarket aisles, such as milk yoghurt (liquid consistency) and buttermilk yoghurt, were also excluded. Excluded items photographed during data collection were deleted prior to data analysis. 

Data was collected during the audit by taking photographs of the front, back, sides, top, and bottom of all eligible yoghurt products within the “dairy”, and “yoghurt” aisles of each supermarket. All eligible yoghurt products available at the time of data collection were audited. Information collected from each yoghurt product included brand name and variety, price, nutrition information panel (NIP) (serving size, energy, protein, fibre, sodium, total fat, saturated fat, sugar, and any other nutrients present on the panel), ingredients list (percentage of fruit, vegetables, nuts and legumes (FVNL), natural and artificial colours, flavours, preservatives, emulsifiers, sweeteners), and the health star rating (HSR) (if available). 

Health, nutrition, and related claims were also collected for each yoghurt product including the number, wording, and type of claim (including nutrition content, high-level health, general-level health, therapeutic, or any other claim which did not fit into the aforementioned category). To ensure that all data was accurately obtained during collection, a cross-check of 10% of the items photographed (e.g., checking the researcher’s phone gallery to ensure that photographs of the products had all the necessary information for analysis) [38]. In cases where products were available in multiple sizes, the largest size was included, as it is more likely to carry the greatest number of claims [39]. Variety yoghurt packs with different flavours were treated as individual products, as their nutritional composition and claims may differ between flavours. All duplicate items were deleted prior to data analysis. 

After data collection, all data was entered into a Microsoft Excel 365 v 16.0 (Microsoft Corporation, Santa Rosa, California, United States, 2017) spreadsheet for analysis. 

### 2.4. Calculation of Nutrient Profiling Scoring Criterion (NPSC) and Health Star Rating (HSR)

Following completion of the audit, the information collected during the audit was used to apply the nutrient profiling scoring criterion (NPSC) for all analysed yoghurt products, in order to determine which products were eligible to carry a health claim [11]. The food properties required for the NPSC were obtained from the NIP of each product, including energy, total and saturated fat, sugar, sodium, protein, fibre, and percentage of fruit, vegetables, nuts and legumes (FVNL) [11].

The NPSC allocates food properties into two categories, baseline points (B) and modifying points (V Points). Baseline points include energy, saturated fat, sugar, and sodium of the product per 100 g, while modifying points consist of the product’s FVNL percentage, protein (P), and fibre (F) per 100 g. The final score is calculated by subtracting the total modifying points (FVNL + P + F) from baseline points; baseline points—((FVNL) + (P) + (F)) [11]. The final value is then utilised to determine whether or not a product is eligible to carry health claims. 

The value used to indicate whether a product meets the NPSC varies across food and beverage categories. Yoghurt, and yoghurt-containing products must achieve a score less than four to meet the NPSC and be eligible to carry a general or high-level health claim [11]. In the current study, while most components required for the NPSC were obtained from the NIP and ingredients list, certain properties such as fibre and saturated fat content were not available on some of the audited products. In such instances, the contents were estimated based on averages of similar yoghurt products containing the food property on the NIP (Supplementary Table S1). Finally, in cases where estimations of nutrient content appeared on a product’s NIP such as “<1 g” or “<0.1 g” the value was assumed to be half of that specified, for instance if the NIP stated “Fibre: <1 g”, it was treated as 0.5 g. Due to the low amount of these nutrients present in the products, it should be noted that this estimation would not affect the NPSC calculated. 

In cases where FVNL percentages were not present on yoghurt packaging, estimates were made based on the ingredient list (which must be listed in descending order according to weight) [40] and similar yoghurt products containing similar ingredient/s. However, it should be noted that in order for a product to be eligible for FVNL points it must contain either 25% or more of concentrated fruit or vegetables; more than 40% nonconcentrated FVNL; or a mixture of more than 40% of nonconcentrated FVNL and concentrated fruit or vegetables [41]. In terms of yoghurt products analysed, concentrated fruit or vegetable content was the most common FVNL criteria assessed, with the majority of products not meeting the cut-off required, suggesting that the imputation of the amount of FVNL would have minimal effect on the NPSC calculated. 

The HSR was also calculated for all yoghurt products. Utilising the nutrition composition per 100 g, the HSR allocates points for energy, saturated fat, sodium, and total sugar contents (risk factors for chronic disease), as well as FVNL content, protein, and fibre [42]. In addition to calculating the HSR for all products, whether a product listed the HSR on its packaging was also noted.

### 2.5. Claim Type and Classification 

#### 2.5.1. Included Claims 

The FSC defines “claims” as “an express or implied statement, representation, design or information in relation to a food or a property of food” [10]. In consequence, the formatting of claims (images, testimonials, or text) on food packaging was considered negligible and all claims were included in this analysis, regardless of their format. 

Claims identified in the audit for analysis were classified in accordance with the FSC, as nutrition-content claims (e.g., “good source of calcium”), general-level health claims (e.g., “probiotics assist with digestion”), high-level health claims (e.g., “high calcium assists in prevention of osteoporosis”), or therapeutic claims (e.g., ”a diet low in sodium could cure high blood pressure”) [10] (Table 2). General health claims that were not pre-approved under Standard 1.2.7, were categorised as self-substantiated claims, in line with the FSC [43]. The HSR, although not outlined in Standard 1.2.7 [10], was also included in analysis due to its increasing prevalence and awareness amongst consumers [44]. 

#### 2.5.2. Excluded Claims

Puffery claims (e.g., “the goodness of milk”, “natural”, “organic”) were excluded from analysis as they were not defined within the FSC (Figure S1). Additionally, endorsements (e.g., “used by the Australian Olympic Team”), while outlined by the FSC, were not included in analysis as they are not considered to constitute a nutrition and health claim per se (Table 2). 

### 2.6. Claim Compliance 

All claims were evaluated for compliance against the FSC and Schedule Four [10]. This included ensuring that products met the NPSC (in the case of health claims) [11] and nutrient quantity levels when claims were related to certain properties of food [45]. For example, a yoghurt product making a claim regarding it being a “good source of protein” must contain at least 10 g of protein per serving. Self-substantiated general-level health claims were checked against the Food Standards Australia New Zealand (FSANZ) Notified Food–Health Relationships database [43] to determine if they had been notified to FSANZ in line with FSC requirements. The number, type, and wording of claims was determined. In line with the requirements of the FSC, in order for a comparative claim (for example “25% less fat than our regular vanilla yoghurt”) to be made by a product, it must have specified the ”reference food” [10]; if not, the claim was considered noncompliant. The calculated and packaged HSR (if applicable) for each product was also determined to investigate the number of products containing the HSR and whether the calculated values matched those on the yoghurt products. 

### 2.7. Other Specifications and Assumptions 

During the process of claim classification and assessing claim compliance, professional judgement was required in some cases to categorise claims and determine their compliance. For example, in cases where claim conditions were not specified by the FSC (for example content claims relating to probiotics or live cultures [45]), claim compliance was assessed utilising criteria outlined in Clause 13 of Standard 1.2.7, which states, “nutrition-content claims can be made about a food property not mentioned in Schedule Four, although the claim may only state if the product does or does not contain the food property, or if the food contains a specified amount of the food property, or a combination of both” [10]. In consequence, yoghurt products including claims on such food properties were only assessed as compliant if they contained permitted statements (e.g., “three probiotics”, “contains live yoghurt cultures”) set out by Clause 13 of Standard 1.2.7. 

Throughout the study, words used to describe properties of yoghurt products such as “rich” and “good” were classified as a ”good source of (nutrient)” in claim analysis. Nutrition-content claims that were not specifically referred to in the FSC such as “gelatine-free” or “no stevia” were classified as “other”. Claims containing a quantity without specifying individual nutrients such as “contains nine essential amino acids” were treated as one nutrition-content claim and classified as “other”. In addition, claims worded as, e.g., “low-fat yoghurt” were evaluated to the same criteria as “low fat” claims. Claims relating to probiotics were classified as “live cultures”, and “thickeners”, “sweeteners”, and ”food acids” were classified as claims relating to artificial colours/flavours/preservatives. 

### 2.8. Data Quality 

After data analysis, a second researcher (EN) conducted a random audit of a 10% subsample of yoghurt products (*n* = 34 products). This involved independently calculating the NPSC and HSR of each product as well as the number, type, and wording of claims on the products sampled, and determining the compliance of these claims. 

### 2.9. Statstical Analysis 

Data was analysed and summarised using Microsoft Excel 365 v 16.0 (Microsoft, 2017). The total number of products surveyed and those carrying claims was determined, as well the average and total number of products from each yoghurt category. Data was also analysed by claim type, with the number and percentage of claims per yoghurt category assessed. Further classification of claim type and yoghurt category was undertaken according to each product’s compliance with the NPSC, to investigate which yoghurt products were able to make health claims, as well as compliance of claims with the NPSC. The presence of the HSR on packaging was classified according to different yoghurt categories, with the average calculated HSR also determined. The compliance of claims was analysed by evaluating the proportion of claims compliant per yoghurt category, claim type (FSC) and FSANZ NPSC.

## 3. Results

Overall, 340 products were surveyed across five supermarkets in the Illawarra region with a total of 1680 health, nutrition, and related claims identified across these products. 

### 3.1. Claim Prevalence

During the process of claim classification and assessing claim compliance, professional judgement was required in some cases to categorise claims and determine their compliance. Of the 13 yoghurt categories eligible for inclusion (Table 1) yoghurt products from 10 categories were identified during the audit. Products under the classification of “yoghurt, unspecified fat”, “yoghurt, added nutrients or other substances”, and “yoghurt, flavoured or added fruit with added cereal, full fat” were not found in the selected supermarkets during the audit. 

Table 3 highlights the prevalence of products and claims found across each yoghurt category (*n* = 10). Of the total products surveyed, yoghurt products classified as “yoghurt pouches” (*n* = 91, 26.8%), “yoghurt, flavoured or added fruit and/or cereal, high fat (> 4 g/100 g fat)” (*n* = 72, 21.2%), and “yoghurt, flavoured or added fruit, reduced fat” (*n* = 58, 17.1%) had the highest prevalence across the five supermarkets. The “yoghurt, natural, reduced fat”, category had the lowest proportion of products analysed (*n* = 6, 1.7%). 

Regarding claim prevalence, 333 of the 340 products analysed contained at least one health, nutrition, and/or related claim (97.9%). The products not carrying any claim (*n* = 7) were all found in the “yoghurt, flavoured or added fruit and/or cereal, high fat (>4 g/100 g fat)” category, with all products in the remaining nine yoghurt categories carrying at least one claim (Table 3). 

Table 4 notes that of the products carrying health and/or nutrition claims (*n* = 333), the average number of claims per product was 5.0, with yoghurt products in the “yoghurt, flavoured or added fruit, low fat or skim, sugar sweetened” category having the highest average number of claims per product (7.7), and products in the “yoghurt, flavoured or added fruit and/or cereal, high fat (>4 g/100 g fat)” category having the lowest (2.6). The yoghurt categories which carried the greatest proportion of health and/or nutrition claims were “yoghurt pouches” (*n* = 565, 33.6%), “yoghurt, flavoured or added fruit, reduced fat” (*n* = 265,15.8%), and “yoghurt, flavoured or added fruit and/or cereal, high fat (>4 g/100 g fat)” (*n* = 169, 10.1%) (Table 4). For products carrying claims, the maximum number of total claims found in any one product was 15, and the minimum was one.

### 3.2. Claim Type and Classification 

Of all the health and nutrition claims found (*n* = 1680), 1577 (93.9%) were nutrition-content claims and 103 (6.1%) were general-level health claims (Table 5). No high-level health claims were identified during the audit. Table 5 identifies the prevalence of the various claim types in the yoghurt categories (*n* = 10) analysed during the audit. The highest proportion of nutrition-content claims per yoghurt category were found in the “children’s yoghurt” (*n* = 21, 100.0%), “yoghurt, natural, skim, and nonfat” (*n* = 66, 100.0%), and “yoghurt, flavoured or added fruit, low fat or skim, sugar sweetened” (*n* = 52, 100.0%) categories, whilst products in the “yoghurt, flavoured or added fruit and/or cereal, high fat (>4 g/100 g fat)” category (*n* = 169) contained the lowest proportion of general-level health claims (*n* = 3, 1.8%) of categories that contained at least one of these claims. 

The five most common types of nutrition content and general-level health claims assessed in the audit are shown in Table 6. The most common nutrition-content claim (*n* = 1577) identified was “no artificial colours/flavours/preservatives” (*n* = 414, 26.3%), followed by claims classed as “other’ (e.g., ”gelatine free”, “no powders”) (*n* = 194, 12.3%) and ”live yoghurt cultures”(*n* = 191, 12.1%). Of the 103 general-level health claims assessed, 29 were classified as “self-substantiated” (*n* = 29, 28.2%), with the majority of these claims relating to digestive health and wellbeing, 23 (22.3%) were classified as “calcium” and 20 (19.4%) were related to “protein”. General-level health claims classified as “not specified” made up 5.8% (e.g.,“Healthy snack that will give you the right fuel to keep going”), which related to claims that did not fall into any one specific general-level claim condition outlined in the FSC. Claims that made up less than six percent of the total nutrition-content claims (e.g., “lactose free” and “source of dietary fibre”) and less than five percent of general-level health claims (e.g., “calcium and vitamin D for strong bones and teeth”) were grouped as “less common claims”.

### 3.3. Claim Classification according to FSANZ NPSC and the Health Star Rating 

Of the 340 products analysed, 277 (81.5%) met the NPSC and were therefore eligible to make a health claim while 63 products (18.5%) did not meet the NPSC and cannot make a health claim under the FSC (however it should be noted that these products are still able to make nutrition-content claims under the FSC regulations). All products (100.0%) in six out of 10 yoghurt categories analysed met the NPSC (Table 7). Of the other categories, the “yoghurt, flavoured or added fruit and/or cereal, high fat (>4 g/100 g fat)” category contained the highest proportion (*n* = 52, 72.2%) of products that did not meet the NPSC.

In addition to examining the proportion of overall products which met the NPSC, individual claims (*n* = 1680) were also explored according to whether the product met the NPSC. According to the FSC, nutrition-content claims (*n* = 1577) are allowed on food packaging regardless of whether the product meets the NPSC, while general-level health claims (*n* = 103) and high-level health claims (none found during analysis) can only be made on products that meet the NPSC. 

Overall, of the 1680 nutrition content and general-level health claims analysed, 1531 (91.1%) were on products that met the NPSC. From the nutrition-content claims analysed (*n* = 1577), 1429 (90.6%) were on products that met the NPSC, while 148 (9.4%) were on products that did not. In regard to general-level health claims (*n* = 103), 102 claims (99.0%) were on products eligible to carry health claims, while only one general-level health claim (1.0%) was on a yoghurt product that did not meet the NPSC, contradictory to violating the FSC regarding the conditions for health-related claims to be made on products. 

The HSR was calculated for all products (*n* = 340) regardless of whether it was available on their packaging. All ratings appearing on yoghurt products matched the calculated HSR for that product. The average calculated HSR was 3.5, with 191 products (56.2%) above the average, and 149 (43.8%) products below. Of the products above the calculated HSR average (*n* = 191), 26 (13.6%) products carried the HSR on their packaging, while 15 of the 149 products (10.1%) below the average carried the HSR on their packaging. Table 8 details the products displaying the HSR on their packaging by yoghurt category. Of the 340 products analysed, 299 (87.9%) did not contain the HSR on the product packaging, while 41 (12.1%) products did contain the HSR. Nine out of the 10 yoghurt categories had at least one product that carried the HSR, with the “yoghurt, flavoured or added fruit, reduced fat” category containing the highest number of products (*n* = 16) carrying the HSR on packaging. The “yoghurt, flavoured or added fruit, low fat or skim, sugar sweetened” category did not contain any products with the HSR on their packaging, with all categories (*n* = 10) containing a higher proportion of products not carrying the HSR.

### 3.4. Claim Compliance 

Of the 1680 claims assessed for claim compliance, 1637 (97.4%) were compliant with the FSC, while 43 claims (2.6%) were not compliant (Table 9). When considered for the 340 products surveyed during the audit, this equated to 297 (87.4%) compliant products (i.e., they contained no noncompliant claims) and 43 (12.6%) noncompliant products (i.e., they contained at least one noncompliant claim). A total of nine out of 10 yoghurt categories assessed contained at least one noncompliant claim; however, all categories had a higher proportion of compliant claims. All claims on products in the “yoghurt, natural, reduced fat” category (*n* = 30) were compliant with the code. The “children’s yoghurt” category had the highest proportion of noncompliant claims (*n* = 2, 9.5%), with the “yoghurt, flavoured or added fruit, full fat” category also carrying a substantial proportion of noncompliant claims (*n* = 13, 8.1%). 

Of all the nutrition-content claims (*n* = 1577), 1543 (97.8%) were compliant with the FSC, while 34 (2.2%) were not compliant with the FSC. Specific nutrition-content claims with the highest proportion of noncompliance included, “low fat” (*n* = 14, 41.2%,), and “reduced/light sugar” (*n* = 12, 35.3%), as they did not meet the quantitative cut-offs required to make these claims under the FSC (for example, products that exceeded the 3g of fat per 100g required to make a low-fat claim). In terms of general-level health claims, which require stricter criteria to be followed by food manufacturers, nine of the 103 claims (8.7%) were noncompliant, while 94 claims (91.3%) were compliant with the code. Of the nine noncompliant general-level health claims, six (66.7%) were classified as “general: not specified” and assessed as noncompliant as they were not a pre-approved claim under the FSC, nor a self-substantiated claim listed on the FSANZ website.

## 4. Discussion

This was the first study to assess the compliance of health, nutrition, and related claims in Australian yoghurt products since the revision of the FSC in 2013 [10]. A total of 340 different yoghurt products were found across the supermarkets audited, with 97.9% containing at least one identified claim. These findings are in contrast with previous Australian research conducted prior to the revision of the FSC, which found that only 30% of products carried health or nutrition claims [22], with most claims compliant with the FSC (97.4%). In the present study, a total of 1680 health and nutrition claims were found across audited products. Of these, 93.9% of claims were classified as nutrition-content claims, with the remainder classified as general-level health claims. Most yoghurt products audited (81.5%) met the nutrient profile scoring criterion (NPSC) and were thus considered to have a healthy nutrient profile, and eligible to carry health claims. Interestingly, this does not align with previous research into other food categories, which found higher proportions of products that did not meet the NPSC [4,27,46]. 

The presence of health and nutrition claims on food packaging has rapidly expanded in the past decade [22] as the widening of permitted claims [10], and influence of claims on consumer purchasing behaviour [47,48], has enticed food manufacturers to take advantage of this potential marketing method [2,47,49]. In the present study, 97.9% of yoghurt products audited contained a health, nutrition, or related claim, with an average of 5.0 claims per yoghurt product. These results exemplify the upward trend in claim prevalence, with previous studies noting substantially less yoghurt and dairy products carrying claims (29–55%) [22,23,50]. Similar results have been found for other food categories such as breakfast cereals [14,51], bakery products, and fruit and vegetable items [6,22]. Altogether, these findings reiterate the dynamic nature of the food environment, and the corresponding potential that food manufacturers see in using health and nutrition claims as a form of promotion and marketing. It is, therefore, imperative that policy developers and health professionals regularly monitor health messaging on product packaging to ensure that information is accurate and transparent for the consumer.

The revised FSC classifies health and nutrition claims into four categories: nutrition-content claims, general-level health claims, high-level health claims, and therapeutic claims (which are not permitted to appear on food labels) [10]. Similar to previous audits of other food categories conducted in Australia [14,16,27], the most common claim type identified was nutrition-content claims (93.9%), with a lower proportion of general-level health claims (6.1%) (Table 5). Interestingly however, no high-level health claims or therapeutic claims were found during the audit, differing to findings seen in other food categories [3,14,16,27].

Greater use of nutrition-content claims compared to general and high-level health claims has previously been reported and is speculated to be due to stricter criteria required to make health claims [16,27,52,53]. The reason for the absence of high-level claims in yoghurt products compared to other food categories may also be due to the varying nutrient profile of yoghurt products. For instance, it was observed that some varieties of plain yoghurt met the minimum quantity of calcium to make high-level health claims (related to calcium), whereas a flavoured variety by the same manufacturer did not contain the minimum quantity of calcium to make a claim. Therefore, if yoghurt manufacturers wanted to make high-level health claims on certain products, they would be required to have substantially different packaging for each flavour profile, which may not be physically and/or economically feasible. 

Previous investigations have, however, seen the benefit in altering packaging to promote claims of this nature, with an Irish study noting that almost 16% of yoghurt products surveyed contained a high-level health claim [50]. Whilst claims of this nature must be accurate, the benefits [54,55] and popularity of dairy yoghurt [46,56] provide health professionals and manufacturers with an opportunity to use these claims to provide crucial health information to consumers. In all, while the absence of therapeutic claims suggests that the FSC is promoting safe and consumer-orientated practices in preventing the use of illegitimate claims [10], manufacturers should be encouraged to evaluate their products to ascertain whether higher-level claims are possible, in order to promote consumption of yoghurt as a core food. 

Nutrition-content claims tend to encompass a range of food properties without as stringent criteria as other claim types, leading to a higher prevalence in the majority of food categories, including yoghurt (Table 5). The most common nutrition-content claim was “no artificial colours/flavours/preservatives” (Table 6), which was also found to be highly prevalent in audits of the claims on other food categories [14,27]. While the FSC outlines criteria for such claims [45], there is relatively limited regulation around factors such as the wording, colour, and position of nutrition-content claims which brings into doubt whether such claims are valuable for guiding consumer choice. It is therefore encouraged that enhanced regulation around the context and wording of nutrition-content claims be initiated to ensure marketing within the food industry does not come at the cost of transparent health information. 

Claims, messaging, and advertising not included in any of the four categories outlined by the FSC, were classified as “puffery claims”, which are vague and exaggerated claims that cannot be quantifiable [56] for example, “natural”, and “boost your daily balance”. While these claims are not included in the FSC, and so were not included for additional analysis in the present study, they were observed to be present in high prevalence in audited yoghurt products, further reinforcing the marketing techniques used by manufacturers to influence consumers. These findings support previous research relating to the high prevalence of marketing on food packaging [16]. Combined with the influence of claims on consumer food choices [47], it is clear that the presence of puffery claims may have significant consequences at the consumer and public health level. Such findings warrant direct consumer research into how puffery claims, and other marketing techniques are received and interpreted, and their impact on consumer expectations and decision making. Additionally, these results further the case for increased regulation around the marketing of food and beverage products, and challenge health professionals, such as dietitians, to accurately decipher and interpret this information in order to empower consumers to make health-conscious decisions.

The NPSC was applied to all products in order to assess whether they were allowed to carry a health claim (general-level or high-level health claim) [11]. Previous research exploring the use of the NPSC and HSR on Australian dairy products not only indicated a high level of agreement between products with a HSR greater than three and NPSC eligibility, but also that 74.0% of yoghurt products met the NPSC [46]. A slightly higher proportion of products meeting the NPSC was found in the current study (*n* = 277, 81.5%); this slight increase may be due to differences in yoghurt products at the time of analysis (Sydney 2014 vs. Illawarra 2020), but overall, these findings reinforce the active response by yoghurt manufacturers to alter and reinvigorate products to adhere to nutrient profiling and enhance product appeal [57]. 

The importance of the NPSC in distinguishing “healthy” and “unhealthy” products was conveyed to varying extents in the current study. While it is important that healthy products are eligible to carry health claims, the NPSC also plays an important role in restricting less healthy products from carrying health-related claims. Although yoghurt is considered to be a core food [58], the ability of the NPSC to distinguish between products was illustrated in the present study when examining the results by product category. For instance, 72.2% of yoghurt products categorised as “yoghurt, flavoured or added fruit and/or cereal, high fat (>4 g/100 g fat)” did not meet the NPSC (Table 7). The higher fat content in the majority of these products, and associated health complications related to elevated consumption of saturated fat [59,60], emphasise the crucial role that the NPSC can play in creating a supportive food environment for consumers through minimising the presence of potentially influential health claims on unhealthy products. 

Despite these benefits, nutrition profiling systems such as the NPSC have been criticised in the literature for their lack of specificity. For instance, in the current model, nutrient assessments and calculations are consistent across all food groups with the only distinction coming in the point-based categories to determine whether products can carry a health claim [61]. While this promotes uniformity and ease-of-use for consumers, some believe that nutrient profile criteria should be different for each core food group [62,63]. The separation of criteria could allow for greater transparency and consistency [62] of foods classified as “healthy” and “unhealthy” under the NPSC. For instance, while over 70% of yoghurt products classified as “high fat” did not meet the NPSC, research suggests that higher dairy fat intake is not associated with the same cardiovascular risks as saturated fat in other foods [64,65]. Furthermore, the average saturated fat content per serving of dairy-yoghurt products analysed was only 2.8 g, highlighting the minimal contribution such products make to consumers’ total daily intake of saturated fat. In all, this emphasises that moving towards a food-group-specific profiling system may not only enhance the validity of the NPSC [62], but also shift focus from viewing foods purely based on their nutrients to a more holistic outlook on food, aligning with the concept of food synergy [66,67,68]. Overall, these findings, along with the lack of specificity in the current NPSC [61] invites re-examination of this nutrient profiling method to ensure that it correctly considers the health value of different foods. 

Another form of nutrient profiling utilised by food manufacturers is the HSR. Introduced in 2014, this voluntary rating system was established to assist consumers make healthier food and beverage choices [69]. While this scheme is not recognised in the revised FSC [10], the increasing prevalence of this label on product packaging [70] and controversial uptake by consumers and manufacturers [71,72] justifies analysis and assessment into this prevailing component of the Australian food environment. In the current study, only a small proportion of yoghurt products (12.1%) carried the HSR. While these findings are consistent with other studies of dairy products [70], the established link between the HSR and NPSC of most categories in promoting foods consistent with the Australian Dietary Guidelines (ADG) (e.g., foods considered “healthy” under the NPSC generally have higher HSR) [46,63] brings into question why yoghurt manufacturers have not followed other food categories [46,73,74] in using the HSR on product packaging. This presents the opportunity for future research to investigate this disparity and provide further clarification into HSR uptake and manufacturer perceptions of the rating system.

In addition, previous studies have shown that a vast majority of consumers see the HSR as an effective and easy-to-use method of quickly evaluating the “healthiness” of products [71]. Food manufacturers in certain food categories have increasingly utilised the HSR [70] in attempts to add appeal to their products, while also potentially promoting an effective means of prompting positive health choices within the food environment. Despite this, progress within this space has been uneven; as noted in the current study where yoghurt products displaying the HSR generally had higher ratings, which is consistent with findings in other food categories [70]. It is therefore recommended that policy developers and health professionals improve the reliability and accuracy of the HSR to be consistent across all food and beverage products, with mandatory HSR labelling required on all products regardless of the rating. 

A crucial aspect required for consumers to navigate the food environment in a health-orientated manner is to ensure that health, nutrition, and related claims are accurate and consistent. This can be achieved through examining the compliance of such claims against relevant legislation, which in Australia is the FSC. Of all the health and nutrition claims analysed in the audit, 97.4% were compliant with the revised FSC (Table 9). The high level of compliance seen in yoghurt claims is consistent with findings in previous audits [14,16,27] of other food categories. This suggests that in the four years since enforcement of the revised FSC, manufacturers have continued to adapt and adhere to regulation regarding the use of health and nutrition claims on product packaging. The high rate of claim compliance among yoghurt products (Table 9) also highlights the effectiveness of the FSC in harnessing product labelling to support consumers to make informed health decisions in the food environment. 

In terms of yoghurt categories assessed, the majority of claims in each category were compliant with the FSC (Table 9). Yoghurt products in the “children’s yoghurt” category had the highest proportion of noncompliant claims (9.5%); however, the small number of children’s yoghurt products assessed (*n* = 19) must be considered when interpreting the data. Nonetheless, the high proportion of noncompliant claims in this category is of some concern, due to the influence of health messaging and marketing on children [16], and preference for “children”-labelled products by parents and carers [75]. Products in the “yoghurt, flavoured or added fruit, full fat” also had a substantial proportion of noncompliant claims (*n* = 13, 8.1%) compared to other categories assessed. The presence of noncompliant claims in these categories could be due to a number of reasons, including misinformed marketing techniques, lack of awareness and understanding of the standards governing health and nutrition claims, as well as possibly an absence of product packaging reformulation prior to the revised FSC. It is therefore suggested that educational seminars in relation to the FSC should be targeted at food manufacturers, as misinformation [76] in combination with additional marketing techniques [2,47,48,49] can promote negative purchasing and health behaviour.

Finally, regarding claim-specific compliance, 97.8% of nutrition-content claims were compliant with the FSC; which, while providing a sense of reassurance, brings into question whether further research might be needed to ensure that these claims are being interpreted as anticipated, or whether revision of their use might be needed regarding the wording and/or marketing of such claims. Moreover, almost one in 10 general-level health claims were noncompliant with the code (8.7%), with only one health claim appearing on a product that did not meet the NPSC. It is pleasing to note, however, that this proportion is substantially lower than findings in other food categories [14,16,27]. This difference may be due to the higher rate of self-substantiated claim compliance in the current study compared to others [14], emphasising manufacturers’ successful utilisation of the protocol under Standard 1.2.7 to promote consumer confidence in a variety of health claims [77]. It is recommended, however, that a greater degree of evaluation and analysis by manufacturers, health professionals, and policy developers is undertaken regarding noncompliant claims and potentially problematic and influential areas such as the wording and requirements of health and nutrition claims. Overall, this will assist in promoting a health-conscious food environment in the absence of inaccurate, ambiguous, and misleading health and nutrition claims, further supporting consumers to make healthy food choices. 

There are a number of limitations that should be considered when interpreting the results of this study. As this study only examined the health, nutrition, and related claims in Australian yoghurt products, results cannot be generalised to all food categories in the Australian food environment. In addition, as all five supermarkets surveyed were in the Illawarra region, future researchers are challenged to examine and assess yoghurt products in other regions of Australia, as availability may vary across locations [78]. This limitation was somewhat mitigated by the selection of supermarkets across a range of socio-economic areas [79], and the choice of supermarket type based on market share [28]. The included and excluded yoghurt products list for the audit (Table 1) was established using the AUSNUT 2011-13 Classification System [36] in conjunction with yoghurt products found in the targeted supermarket aisles (the ”dairy” and/or “yoghurt” aisle) (see Methods). Of the 13 yoghurt categories included for analysis, only 10 were identified during the audit, which may have been due to unavailability at the time of surveying, products moved to other aisles of the supermarket and/or human error in category allocation. Future research may address these limitations by repeating the audit to include more supermarket aisles and stores. In addition, while nondairy yoghurts appeared within the targeted supermarket aisles, they were not outlined in the “yoghurt” category of the AUSNUT Classification System [36]. As these products were classified as “milk substitutes” and this study focused on dairy yoghurts, nondairy yoghurts were excluded from analysis. However, as these products are gaining traction and popularity among consumers [80], investigating the compliance of claims and consumer interpretation within this category in future research may be beneficial in further understanding the food environment.

Finally, it should be noted that as the FSC is not intended to be prescriptive, claim compliance and classification was at times ambiguous, and in such cases a second researcher was consulted (EN), and interpretations of the revised FSC were made. As such, it should be noted that findings may be categorised differently among other researchers, manufacturers, and regulators of the code. 

## 5. Conclusions

This study, which was the first to assess the compliance of health, nutrition, and related claims in Australian yoghurt products since the revision of the FSC in 2013, identified that dairy-yoghurt products had a high prevalence of claims (*n* = 1680), with nutrition-content claims the most common (93.9%). Most claims assessed were compliant with the code (97.4%), reinforcing manufacturers and policy developers’ successful enforcement of the revised code. Although most claims were compliant, the greater proportion of noncompliant claims in the general-level health claims category shows the need for continued adaptation and monitoring by food businesses and authorities to ensure that health, nutrition, and related claims on product packaging are accurate, transparent, and understandable.

As a whole, the NPSC was a suitable profiling system in distinguishing “healthy” and “unhealthy” yoghurt products, with a high proportion of higher fat products unable to carry health claims. However, the inability of the NPSC to distinguish between food groups and/or categories despite varying evidence regarding the effect of nutrients in certain products, reinforces the need for nutrient profiling methods to adapt in order to remain relevant and effective. Moreover, the low proportion of yoghurt products carrying the HSR potentially underscores the consumer and manufacturer uncertainty in relation to the use and importance of this rating scheme. Nonetheless, the potential and increasing prevalence of the HSR among other food categories highlights how the HSR could be employed on yoghurt packaging to promote health-orientated practices and choices within the food environment. 

Additional research into the various marketing techniques used by food manufacturers is warranted from findings in this study, as of the observed use of puffery claims indicates that this needs to be researched further to ensure these are not unduly influencing consumer behaviour. Furthermore, the ambiguity of wording and context of health, nutrition, and related claims challenges health professionals and policy developers to evaluate and bring in stricter measures to promote consistency in health messaging. Overall, through enhanced monitoring and collaboration between food manufacturers, policy developers and health professionals, health, nutrition, and related claims can be utilised to support the food industry in promoting health-orientated practices and food choices among consumers. 

## Figures and Tables

**Table 1:** Inclusion and exclusion list of analysed yoghurt products (based on AUSNUT 2011-13 categorisation).

Included Products	Excluded Products
Yoghurt, natural, regular fat, and high fat (>4 g/100 g fat)	Frozen yoghurt (desserts)
Yoghurt, natural, reduced fat	Yoghurt-based Confectionary
Yoghurt, natural, skim and nonfat	Infant Yoghurts/Custards
Yoghurt, flavoured or added fruit and/or cereal, high fat (>4 g/100 g fat)	Nondairy-based yoghurt products
Yoghurt, flavoured or added fruit, full fat	Milk yoghurt (liquid consistency)
Yoghurt, flavoured or added fruit with added cereal, full fat	Buttermilk yoghurt
Yoghurt, flavoured or added fruit, reduced fat	
Yoghurt, flavoured or added fruit, low fat or skim, sugar sweetened	
Yoghurt, flavoured or added fruit, low fat, or skim, intense sweetened	
Yoghurt, added nutrients or other substances	
Yoghurt, unspecified fat	
Children’s yoghurt	
Yoghurt pouches	

**Table 2:** Inclusion and exclusion list of analysed claims.

Included Claims	Excluded Claims
Nutrition content claims	Puffery claims
General-level health claims (pre-approved and self-substantiated)	Endorsements
High-level health claims	
Therapeutic claims	

**Table 3:** Number and proportion of claims prevalent across yoghurt categories.

Yoghurt Category	Product Category		Products Carrying Claims	
	(*n*)	% of Total Products Surveyed	(*n*)	% of Products per Category Carrying Claims
Yoghurt, natural, regular fat, and high fat (>4 g/100 g fat)	30	8.8%	30	100.0%
Yoghurt, natural, reduced fat	6	1.7%	6	100.0%
Yoghurt, natural, skim and nonfat	10	2.9%	10	100.0%
Yoghurt, flavoured or added fruit and/or cereal, high fat (>4 g/100 g fat)	72	21.2%	65	90.3%
Yoghurt, flavoured or added fruit, full fat	30	8.8%	30	100.0%
Yoghurt, flavoured or added fruit, reduced fat	58	17.1%	58	100.0%
Yoghurt, flavoured or added fruit, low fat or skim, sugar sweetened	7	2.1%	7	100.0%
Yoghurt, flavoured or added fruit, low fat, or skim, intense sweetened	28	8.2%	28	100.0%
Children’s yoghurt	8	2.4%	8	100.0%
Yoghurt pouches	91	26.8%	91	100.0%
**Overall**	**340**	**100.0%**	**333**	**n/a**

**Table 4:** Total and average numbers of health, nutrition, and related claims per yoghurt category.

Yoghurt Category	Total Number of Claims		Average Claims per Product
	(*n*)	% of All Claims	(*n*)
Yoghurt, natural, regular fat, and high fat (>4 g/100 g fat)	167	9.9%	5.6
Yoghurt, natural, reduced fat	30	1.8%	5.0
Yoghurt, natural, skim and nonfat	66	3.9%	6.6
Yoghurt, flavoured or added fruit and/or cereal, high fat (>4 g/100 g fat)	169	10.1%	2.6
Yoghurt, flavoured or added fruit, full fat	161	9.6%	5.4
Yoghurt, flavoured or added fruit, reduced fat	265	15.8%	4.6
Yoghurt, flavoured or added fruit, low fat or skim, sugar sweetened	52	3.1%	7.7
Yoghurt, flavoured or added fruit, low fat, or skim, intense sweetened	184	10.9%	6.6
Children’s yoghurt	21	1.3%	2.6
Yoghurt pouches	565	33.6%	6.2
**Overall**	**1680**	**100.0%**	**5.0**

**Table 5:** Prevalence of claim types per yoghurt category.

Yoghurt Category	Nutrition-Content Claims		General-Level Health Claims		Total
	(*n*)	% of Claims per Yoghurt Category	(*n*)	% of Claims per Yoghurt Category	
Yoghurt, natural, regular fat, and high fat (>4 g/100 g fat)	157	94.0%	10	6.0%	167
Yoghurt, natural, reduced fat	28	93.3%	2	6.7%	30
Yoghurt, natural, skim and nonfat	66	100.0%	0	0%	66
Yoghurt, flavoured or added fruit and/or cereal, high fat (>4 g/100 g fat)	166	98.2%	3	1.8%	169
Yoghurt, flavoured or added fruit, full fat	149	92.6%	12	7.4%	161
Yoghurt, flavoured or added fruit, reduced fat	255	96.2%	10	3.8%	265
Yoghurt, flavoured or added fruit, low fat or skim, sugar sweetened	52	100.0%	0	0%	52
Yoghurt, flavoured or added fruit, low fat, or skim, intense sweetened	171	92.9%	13	7.1%	184
Children’s yoghurt	21	100.0%	0	0%	21
Yoghurt pouches	512	90.6%	53	9.4%	565
**Overall**	**1577**	**93.9%**	**103**	**6.1%**	**1680**

**Table 6:** Most popular health, nutrition, or related claims per claim type.

Nutrition-Content Claims			General-Level Health Claims		
	(*n*)	% of Claim Type		(*n*)	% of Claim Type
Content: no artificial colours/flavours/preservatives	414	26.3%	General: self-substantiated	29	28.2%
Other	194	12.3%	General: calcium	23	22.3%
Content: live yoghurt cultures	191	12.1%	General: protein	20	19.4%
Content: no added sugar	125	7.9%	General: live yoghurt cultures	11	10.7%
Content: gluten free	124	7.8%	General: vitamin B12	6	5.8%
Content: source of calcium	102	6.5%	General: not specified	6	5.8%
Less common claims	427	27.1%	Less common claims	8	7.8%
**Total**	**1577**			**103**	

**Table 7:** Proportion of products that met the NPSC per yoghurt category.

Yoghurt Category	Meets the NPSC		Does not Meet NPSC		Total
	(*n*)	% of Products that met NPSC	(*n*)	% of Products that did not Meet the NPSC	
Yoghurt, natural, regular fat, and high fat (>4 g/100 g fat)	21	70.0%	9	30.0%	30
Yoghurt, natural, reduced fat	6	100.0%	0	0%	6
Yoghurt, natural, skim and nonfat	10	100.0%	0	0%	10
Yoghurt, flavoured or added fruit and/or cereal, high fat (>4 g/100 g fat)	20	27.8%	52	72.2%	72
Yoghurt, flavoured or added fruit, full fat	29	96.7%	1	3.3%	30
Yoghurt, flavoured or added fruit, reduced fat	58	100.0%	0	0%	58
Yoghurt, flavoured or added fruit, low fat or skim, sugar sweetened	7	100.0%	0	0%	7
Yoghurt, flavoured or added fruit, low fat, or skim, intense sweetened	28	100.0%	0	0%	28
Children’s yoghurt	8	100.0%	0	0%	8
Yoghurt pouches	90	98.9%	1	1.1%	91
**Overall**	**277**	**81.5%**	**63**	**18.5%**	**340**

**Table 8:** Number of yoghurt products carrying the HSR per yoghurt category.

Yoghurt Category	HSR on Packaging				Total
	YES		NO		
	(*n*)	% of Yoghurt Category that Carried HSR on Packaging	(*n*)	% of Yoghurt Category that did not carry HSR on Packaging	
Yoghurt, natural, regular fat, and high fat (>4 g/100g fat)	4	13.3%	26	86.7%	30
Yoghurt, natural, reduced fat	2	33.3%	4	66.7%	6
Yoghurt, natural, skim and nonfat	1	10.0%	9	90.0%	10
Yoghurt, flavoured or added fruit and/or cereal, high fat (>4 g/100 g fat)	1	1.4%	71	98.6%	72
Yoghurt, flavoured or added fruit, full fat	1	3.3%	29	96.7%	30
Yoghurt, flavoured or added fruit, reduced fat	16	27.6%	42	72.4%	58
Yoghurt, flavoured or added fruit, low fat or skim, sugar sweetened	0	0%	7	100.0%	7
Yoghurt, flavoured or added fruit, low fat, or skim, intense sweetened	6	21.4%	22	78.6%	28
Children’s yoghurt	3	37.5%	5	62.5%	8
Yoghurt pouches	7	7.7%	84	92.3%	91
**Overall**	**41**	**12.1%**	**299**	**87.9%**	**340**

**Table 9:** Compliance of health, nutrition, and related claims per yoghurt category.

Yoghurt Category	Compliant		Noncompliant		Total
	(*n*)	% of Compliant Claims in Each Yoghurt Category	(*n*)	% of Noncompliant Claims in Each Yoghurt Category	
Yoghurt, natural, regular fat, and high fat (>4 g/100 g fat)	165	98.8%	2	1.2%	167
Yoghurt, natural, reduced fat	30	100.0%	0	0%	30
Yoghurt, natural, skim and nonfat	65	98.5%	1	1.5%	66
Yoghurt, flavoured or added fruit and/or cereal, high fat (>4 g/100 g fat)	164	97.0%	5	3.0%	169
Yoghurt, flavoured or added fruit, full fat	148	91.9%	13	8.1%	161
Yoghurt, flavoured or added fruit, reduced fat	263	99.2%	2	0.8%	265
Yoghurt, flavoured or added fruit, low fat or skim, sugar sweetened	50	96.2%	2	3.8%	52
Yoghurt, flavoured or added fruit, low fat, or skim, intense sweetened	179	97.3%	5	2.7%	184
Children’s yoghurt	19	90.5%	2	9.5%	21
Yoghurt pouches	554	98.1%	11	1.9%	565
**Overall**	**1637**	**97.4%**	**43**	**2.6%**	**1680**

## Data Availability

The data presented in this study are available on request from the corresponding author.

## Supplementary Materials

The following are available online at https://www.mdpi.com/article/10.3390/nu13061835/s1, Figure S1: Flow chart of claims included and excluded within the revised FSC (derived from FSANZ Standard 1.2.7), Table S1: Nutrient Estimation Table (Fibre).
