# Supplementary material for: A Cross-Sectional Audit of Nutrition and Health Claims on Dairy Yoghurts in Supermarkets of the Illawarra Region of New South Wales, Australia
Source: Nutrients. 2021 May 27;13(6):1835. doi: 10.3390/nu13061835 (PMC8229526; doi:10.3390/nu13061835)

**Supplementary Material:**

**Table S1 – Nutrient Estimation Table (Fibre)**

**Fibre Content (/100g)**

| | | | |
|---|---|---|---|
| Strawberry | 0.8 | Coconut | 0.1 |
| Mixed Berry | 0.6 | Boysenberry | 0.5 |
| Passionfruit | 0.6 | Evia | 1 |
| Mango | 0.3 | Apple and Cinnamon | 0.6 |
| Raspberry | 0.5 | Honey | 0 |
| Peach and Mango | 1.2 | Almond Coco Loco | 1.4 |
| Vanilla | 0.4 | Cacao | 0 |
| Banana | 0.4 | Coffee | 0 |
| Strawberry and Raspberry | 0.99 | Lemon | 0.3 |
| Natural | 0.1 | Banana and Caramel | 0.5 |
| Blueberry | 0.5 | Dark Cherry | 0.6 |
| Strawberry and Banana | 0.2 | Black Cherry | 0.6 |
| Peach | 0.3 | Fruit Salad | 0.3 |

Supplementary Material

Figure S1: Flow chart of claims included and excluded within the revised FSC(derived from FSANZ Standard 1.2.7)

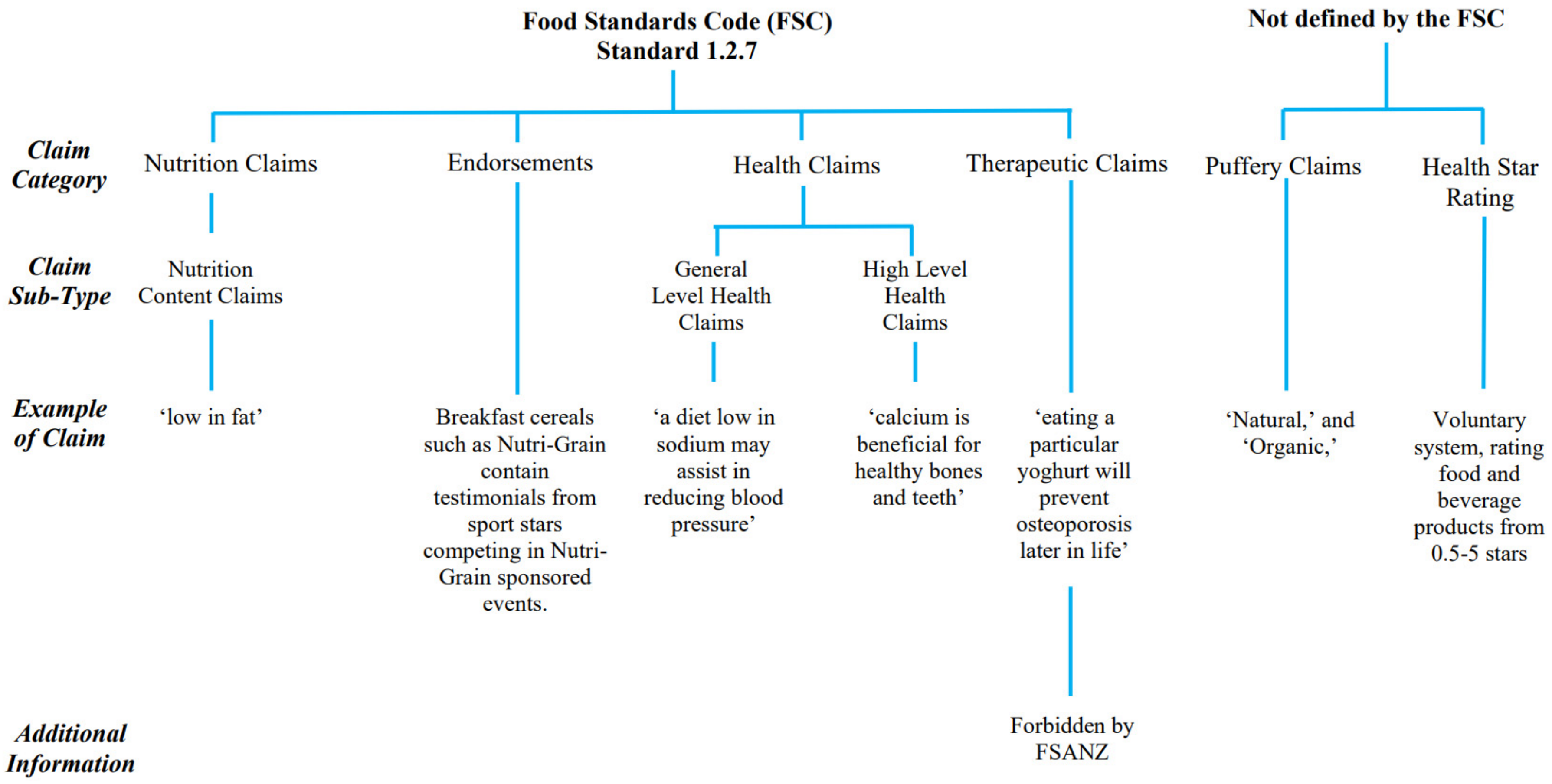

Supplement: Supplementary file 1 [file nutrients-13-01835-s001.zip › nutrients-1246939-supplementary.pdf]
